# Current Awareness on Comparative and Functional Genomics

**DOI:** 10.1002/cfg.421

**Published:** 2005-06


**Comparative and Functional Genomics**
*Comp Funct Genom* 2005; **6**: 259–266
Published online in Wiley InterScience (www.interscience.wiley.com). **DOI:** 10.1002cfg.421

### Current awareness on comparative and functional genomics

CURRENT AWARENESS - USE THE LINKS ONLINE

View this bibliography online at WILEY InterScience®

www.interscience.wiley.com

Your gateway to Wiley journals online

The articles use crossref, the innovative multi-publisher reference linking. This enables readers to move seamlessly from the reference to the cited publication, typically located on a different server and published by a different publisher. Links to MEDLINE abstracts are also available for key journals in the biosciences.

2745

### 1 Reviews & symposia

2004. Special issue: Genomes and evolution. *Curr Opin Genet Dev* **14:** (6)

2004. Special issue: Proceedings of the First Annual Conference of the MidSouth Computational Biology and Bioinformatics Society, November 13-15 2003, Little Rock, Ar, USA. *DNA Cell Biol* **23:** (10)

2004. Special issue: Linking Functional Genomics with Physiology for Global Change Research. *Field Crop Res* **90:** (1)

Bar-Joseph Z. 2004. Analyzing time series gene expression data (Review). *Bioinformatics* **20:** (16) 2493.

Bennetzen JL, Coleman C, Liu RY, Ma JX, Ramakrishna W. 2004. Consistent over-estimation of gene number in complex plant genomes. *Curr Opin Plant Biol* **7:** (6) 732.

Burt DW. 2004. The chicken genome and the developmental biologist. *Mech Dev* **121:** (9) 1129.

Causler B. 2004. Studying the interactome with the yeast two-hybrid system and mass spectrometry. *Mass Spectrom Rev* **23:** (5) 350.

Chignard N, Beretta L. 2004. Proteomics for hepatocellular carcinoma marker discovery. *Gastroenterology* **127:** (5 Suppl 1) S120.

Darvas F, Dorman G, Krajcsi P, Puskas LG, Kovari Z, Lorincz Z, Urge L. 2004. Recent advances in chemical genomics. *Curr Med Chem* **11:** (23) 3119.

Dharmadi Y, Gonzalez R. 2004. DNA microarrays: Experimental issues, data analysis, and application to bacterial systems (Review). *Biotechnol Prog* **20:** (5) 1309.

Engstrom Y, Loseva O, Theopold U. 2004. Proteomics of the *Drosophila* immune response (Review). *Trends Biotechnol* **22:** (11) 600.

Eyers L, George I, Schuler L, Stenuit B, Agathos SN, El Fantroussi S. 2004. Environmental genomics: Exploring the unmined richness of microbes to degrade xenobiotics (Review). *Appl Microbiol Biotechnol* **66:** (2) 123.

Fey MF. 2004. Genomics and proteomics: Expression arrays in clinical oncology. *Ann Oncol* **15:** (Suppl 4) iv163.

Friedman A, Perrimon N. 2004. Genome-wide high-throughput screens in functional genomics. *Curr Opin Genet Dev* **14:** (5) 470.

Guenet JL. 2004. Chemical mutagenesis of the mouse genome: An overview. *Genetica* **122:** (1) 9.

Hachey DL, Chaurand P. 2004. Proteomics in reproductive medicine: The technology for separation and identification of proteins. *J Reprod Immunol* **63:** (1) 61.

Haraguchi N, Inoue H, Mimori K, Tanaka F, Utsunomiya T, Yoshikawa K, Mori M. 2004. Analysis of gastric cancer with cDNA microarray. *Cancer Chemother Pharmacol* **54:** (Suppl 1) S21.

Haubold B, Wiehe T. 2004. Comparative genomics: Methods and applications (Review). *Naturwissenschaften* **91:** (9) 405.

Kabuyama Y, Resing KA, Ahn NG. 2004. Applying proteomics to signaling networks. *Curr Opin Genet Dev* **14:** (5) 492.

Korpelainen H. 2004. The evolutionary processes of mitochondrial and chloroplast genomes differ from those of nuclear genomes (Review). *Naturwissenschaften* **91:** (11) 505.

Kramer R, Cohen D. 2004. Functional genomics to new drug targets. *Nat Rev Drug Discov* **3:** (11) 965.

Lee JS, Thorgeirsson SS. 2004. Genome-scale profiling of gene expression in hepatocellular carcinoma: Classification, survival prediction, and identification of therapeutic targets. *Gastroenterology* **127:** (5 Suppl 1) S51.

Leitner A, Lindner W. 2004. Current chemical tagging strategies for proteome analysis by mass spectrometry (Review). *J Chromatogr B* **813:** (1-2) 1.

Lenz HJ. 2004. Pharmacogenomics and colorectal cancer. *Ann Oncol* **15:** (Suppl 4) iv173.

Liew CC, Dzau VJ. 2004. Molecular genetics and genomics of heart failure. *Nat Rev Genet* **5:** (11) 811.

Llinas M, DeRisi JL. 2004. Pernicious plans revealed: *Plasmodium falciparum* genome wide expression analysis. *Curr Opin Microbiol* **7:** (4) 382.

Lundstrom K. 2004. Structural genomics on membrane proteins: Mini review. *Comb Chem High Throughput Scr* **7:** (5) 431.

Mestres J. 2004. Computational chemogenomics approaches to systematic knowledge-based drug discovery. *Curr Opin Drug Discov Dev* **7:** (3) 304.

Motyka SA, Englund PT. 2004. RNA interference for analysis of gene function in trypanosomatids. *Curr Opin Microbiol* **7:** (4) 362.

Muller-Hagen G, Beinert T, Sommer A. 2004. Aspects of lung cancer gene expression profiling. *Curr Opin Drug Discov Dev* **7:** (3) 290.

O'Donnell R, Holland JW, Deeth HC, Alewood P. 2004. Milk proteomics (Review). *Int Dairy J* **14:** (12) 1013.

Parsons L, Orban J. 2004. Structural genomics and the metabolome: Combining computational and NMR methods to identify target ligands. *Curr Opin Drug Discov Dev* **7:** (1) 62.

Penkett CJ, Bahler J. 2004. Navigating public microarray databases (Review). *Comp Funct Genom* **5:** (6-7) 471.

Prentice H, Webster KA. 2004. Genomic and proteomic profiles of heart disease (Review). *Trends Cardiovasc Med* **14:** (7) 282.

Preuss TM, Caceres M, Oldham MC, Geschwind DH. 2004. Human

In order to keep subscribers up-to-date with the latest developments in their field, this current awareness service is provided by John Wiley & Sons and contains newly-published material on comparative and functional genomics. Each bibliography is divided into 16 sections. 1 Reviews & symposia; 2 General; 3 Large-scale sequencing and mapping; 4 Evolutionary genomics; 5 Comparative genomics; 6 Pathways, gene families and regulons; 7 Pharmacogenomics; 8 EST, cDNA and other clone resources; 9 Functional genomics; 10 Transcriptomics; 11 Proteomics; 12 Protein structural genomics; 13 Metabolomics; 14 Genomic approaches to development; 15 Technological advances; 16 Bioinformatics. Within each section, articles are listed in alphabetical order with respect to author. If, in the preceding period, no publications are located relevant to any one of these headings, that section will be omitted.

Copyright © 2005 John Wiley & Sons, Ltd.

brain evolution: Insights from microarrays. *Nat Rev Genet* **5:** (11) 850.

Rupp S. 2004. Proteomics on its way to study host-pathogen interaction in *Candida albicans*. *Curr Opin Microbiol* **7:** (4) 330.

Scobioala S, Klocke R, Michel G, Kuhlmann M, Nikol S. 2004. Proteomics: State of the art and its application in cardiovascular research. *Curr Med Chem* **11:** (24) 3203.

Sellheyer K, Belbin TJ. 2004. DNA microarrays: From structural genomics to functional genomics. The applications of gene chips in dermatology and dermatopathology. *J Am Acad Dermatol* **51:** (5) 681.

Simillion C, Vandepoele K, Van de Peer Y. 2004. Recent developments in computational approaches for uncovering genomic homology. *Bioessays* **26:** (11) 1225.

Stathopoulos A, Levine M. 2004. Whole-genome analysis of *Drosophila* gastrulation. *Curr Opin Genet Dev* **14:** (5) 477.

Stevens EV, Posadas EM, Davidson B, Kohn EC. 2004. Proteomics in cancer. *Ann Oncol* **15:** (Suppl 4) iv167.

Thiede B, Rudel T. 2004. Proteome analysis of apoptotic cells. *Mass Spectrom Rev* **23:** (5) 333.

Williams K, Wu T, Colangelo C, Nairn AC. 2004. Recent advances in neuroproteomics and potential application to studies of drug addiction. *Neuropharmacology* **47:** (Suppl) 148.

### 3 Large-scale sequencing and mapping

Baliga NS, Bonneau R, Facciotti MT, Pan M, Glusman G, Deutsch EW, Shannon P, Chiu YL, Gan RR, Hung PL *et al*. 2004. Genome sequence of *Haloarcula marismortui*: A halophilic archaeon from the Dead Sea. *Genome Res* **14:** (11) 2221.

Costantini M. 2004. An analysis of sponge genomes. *Gene* **342:** (2) 321.

Fu Y, Hsia AP, Guo L, Schnable PS. 2004. Types and frequencies of sequencing errors in methyl-filtered and high $C_0t$ maize genome survey sequences. *Plant Physiol* **135:** (4) 2040.

Hendrickson EL, Kaul R, Zhou Y, Bovee D, Chapman P, Chung J, De Macario EC, Dodsworth JA, Gillett W, Graham DE *et al*. 2004. Complete genome sequence of the genetically tractable hydrogenotrophic methanogen *Methanococcus maripaludis*. *J Bacteriol* **186:** (20) 6956.

Ihara N, Takasuga A, Mizoshita K, Takeda H, Sugimoto M, Mizoguchi Y, Hirano T, Itoh T, Watanabe T, Reed KM *et al*. 2004. A comprehensive genetic map of the cattle genome based on 3802 microsatellites. *Genome Res* **14:** (10A) 1987.

Messing J, Bharti AK, Karlowski WM, Gundlach H, Kim HR, Yu Y, Wei FS, Fuks G, Soderlund CA, Mayer KFX *et al*. 2004. Sequence composition and genome organization of maize. *Proc Natl Acad Sci U S A* **101:** (40) 14349.

Monosi B, Wisser RJ, Pennill L, Hulbert SH. 2004. Full-genome analysis of resistance gene homologues in rice. *Theor Appl Genet* **109:** (7) 1434.

Monteiro-Vitorello CB, Camargo LEA, Van Sluys MA, Kitajima JP, Truffi D, Do Amaral AM, Harakava R, De Oliveira JCF, Wood D, De Oliveira MC *et al*. 2004. The genome sequence of the Gram-positive sugarcane pathogen *Leifsonia xyli* subsp xyli. *Mol Plant Microbe Interact* **17:** (8) 827.

Munkvold JD, Greene RA, Bermudez-Kandianis CE, La Rota CM, Edwards H, Sorrells SF, Dake T, Benscher D, Kantety R, Linkiewicz AM *et al*. 2004. Group 3 chromosome bin maps of wheat and their relationship to rice chromosome 1. *Genetics* **168:** (2) 639.

Wallis JW, Aerts J, Groenen MAM, Crooijmans RPMA, Layman D, Graves TA, Scheer DE, Kremitzki C, Fedele MJ, Mudd NK *et al*. 2004. A physical map of the chicken genome. *Nature* **432:** (7018) 761.

Xia QY, Zhou ZY, Lu C, Cheng DJ, Dai FY, Li B, Zhao P, Zha XF, Cheng TC, Chai CL *et al*. 2004. A draft sequence for the genome of the domesticated silkworm (*Bombyx mori*). *Science* **306:** (5703) 1937.

Zhou SG, Kile A, Kvikstad E, Bechner M, Severin J, Forrest D, Runnheim R, Churas C, Anantharaman TS, Myler P *et al*. 2004. Shotgun optical mapping of the entire *Leishmania major* Friedlin genome. *Mol Biochem Parasitol* **138:** (1) 97.

### 4 Evolutionary genomics

Bengtsson BO. 2004. Modelling the evolution of genomes with integrated external and internal functions. *J Theor Biol* **231:** (2) 271.

Drnevich JM, Reedy MM, Ruedi EA, Rodriguez-Zas S, Hughes KA. 2004. Quantitative evolutionary genomics: Differential gene expression and male reproductive success in *Drosophila melanogaster*. *Proc R Soc Lond B Biol Sci* **271:** (1554) 2267.

Gao LZ, Innan H. 2004. Very low gene duplication rate in the yeast genome. *Science* **306:** (5700) 1367.

Hillier LW, Miller W, Birney E, Warren W, Hardison RC, Ponting CP, Bork P, Burt DW, Groenen MAM, Delany ME *et al*. 2004. Sequence and comparative analysis of the chicken genome provide unique perspectives on vertebrate evolution. *Nature* **432:** (7018) 695.

Kim KJ, Lee HL. 2004. Complete chloroplast genome sequences from Korean ginseng (*Panax schinseng* Nees) and comparative analysis of sequence evolution among 17 vascular plants. *DNA Res* **11:** (4) 247.

Ovcharenko I, Stubbs L, Loots GG. 2004. Interpreting mammalian evolution using *Fugu* genome comparisons. *Genomics* **84:** (5) 890.

Price AL, Eskin E, Pevzner PA. 2004. Whole-genome analysis of Alu repeat elements reveals complex evolutionary history. *Genome Res* **14:** (11) 2245.

Stankiewicz P, Shaw CJ, Withers M, Inoue K, Lupski JR. 2004. Serial segmental duplications during primate evolution result in complex human genome architecture. *Genome Res* **14:** (11) 2209.

### 5 Comparative genomics

Blakesley RW, Hansen NF, Mullikin JC, Thomas PJ, McDowell JC, Maskeri B, Young AC, Benjamin B, Brooks SY, Coleman BI *et al*. 2004. An intermediate grade of finished genomic sequence suitable for comparative analyses. *Genome Res* **14:** (11) 2235.

Clifton SW, Minx P, Fauron CMR, Gibson M, Allen JO, Sun H, Thompson M, Barbazuk WB, Kanuganti S, Tayloe C *et al*. 2004. Sequence and comparative analysis of the maize NB mitochondrial genome. *Plant Physiol* **136:** (3) 3486.

Conley EJ, Nduati V, Gonzalez-Hernandez JL, Mesfin A, Trudeau-Spanjers M, Chao S, Lazo GR, Hummel DD, Anderson OD, Qi LL *et al*. 2004. A 2600-locus chromosome bin map of wheat homoeologous group 2 reveals interstitial gene-rich islands and colinearity with rice. *Genetics* **168:** (2) 625.

Fukuchi S, Nishikawa K. 2004. Estimation of the number of authentic orphan genes in bacterial genomes. *DNA Res* **11:** (4) 219.

Glockner G, Lehmann R, Romualdi A, Pradella S, Schulte-Spechtel U, Schilhabel M, Wilske B, Suhnel J, Platzer M. 2004. Comparative analysis of the *Borrelia garinii* genome. *Nucleic Acids Res* **32:** (20) 6038.

Huminiecki L, Wolfe KH. 2004. Divergence of spatial gene expression profiles following species-specific gene duplications in human and mouse. *Genome Res* **14:** (10A) 1870.

Kasukawa T, Katayama S, Kawaji H, Suzuki H, Hume DA, Hayashizaki Y. 2004. Construction of representative transcript and protein sets of human, mouse and rat as a platform for their transcriptome and proteome analysis. *Genomics* **84:** (6) 913.

Kovacs GM, Davison AJ, Zakhartchouk AN, Harrach B. 2004. Analysis of the first complete genome sequence of an Old World monkey adenovirus reveals a lineage distinct from the six human adenovirus species. *J Gen Virol* **85:** (10) 2799.

Lai JS, Dey N, Kim CS, Bharti AK, Rudd S, Mayer KFX, Larkins BA, Becraft P, Messing J. 2004. Characterization of the maize endosperm transcriptome and its comparison to the rice genome. *Genome Res* **14:** (10A) 1932.

Linkiewicz AM, Qi LL, Gill BS, Ratnasiri A, Echalier B, Chao S, Lazo GR, Hummel DD, Anderson OD, Akhunov ED *et al*. 2004. A 2500-locus bin map of wheat homoeologous group 5 provides insights on gene distribution and colinearity with rice. *Genetics* **168:** (2) 665.

Masood MS, Nishikawa T, Fukuoka S, Njenga PK, Tsudzuki T, Kadowaki K. 2004. The complete nucleotide sequence of wild rice (*Oryza nivara*) chloroplast genome: First genome wide comparative sequence analysis of wild and cultivated rice. *Gene* **340:** (1) 133.

McLeod MP, Qin X, Karpathy SE, Gioia J, Highlander SK, Fox GE, McNeill TZ, Jiang HY, Muzny D, Jacob LS *et al*. 2004. Complete genome sequence of *Rickettsia typhi* and comparison with sequences of other Rickettsiae. *J Bacteriol* **186:** (17) 5842.

Miyake T, Amemiya CT. 2004. BAC libraries and comparative genomics of aquatic chordate species. *Comp Biochem Physiol C Pharmacol Toxicol* **138:** (3) 233.

Monastyrskaya G, Fushan A, Abaev I, Filyukova O, Kostina M, Pecherskih E, Sverdlov E. 2004. Genome-wide comparison reveals great inter- and intraspecies variability in *B. pseudomallei* and *B. mallei* pathogens. *Res Microbiol* **155:** (9) 781.

Moran G, Stokes C, Thewes S, Hube B, Coleman DC, Sullivan D. 2004. Comparative genomics using *Candida albicans* DNA microarrays re-

Copyright © 2005 John Wiley & Sons, Ltd.

veals absence and divergence of virulence-associated genes in *Candida dubliniensis*. *Microbiology* **150:** (10) 3363.
Moran MA, Buchan A, Gonzalez JM, Heidelberg JF, Whitman WB, Kiene RP, Henriksen JR, King GM, Belas R, Fuqua C *et al*. 2004. Genome sequence of *Silicibacter pomeroyi* reveals adaptations to the marine environment. *Nature* **432:** (7019) 910.
Ong C, Ooi CH, Wang DL, Chong HL, Ng KC, Rodrigues F, Lee MA, Tan P. 2004. Patterns of large-scale genomic variation in virulent and avirulent *Burkholderia* species. *Genome Res* **14:** (11) 2295.
Swigonova Z, Lai JS, Ma JX, Ramakrishna W, Llaca V, Bennetzen JL, Messing J. 2004. Close split of sorghum and maize genome progenitors. *Genome Res* **14:** (10A) 1916.
Zhao SY, Shetty J, Hou LH, Delcher A, Zhu BL, Osoegawa K, De Jong P, Nieman WC, Strausberg RL, Fraser CM. 2004. Human, mouse, and rat genome large-scale rearrangements: Stability *versus* speciation. *Genome Res* **14:** (10A) 1851.
Zhou S, Kile A, Bechner M, Place M, Kvikstad E, Deng W, Wei J, Severin J, Runnheim R, Churas C *et al*. 2004. Single-molecule approach to bacterial genomic comparisons *via* optical mapping. *J Bacteriol* **186:** (22) 7773.

### 7 Pharmacogenomics

Alexander H, Stegner AL, Wagner-Mann C, Du Bois GC, Alexander S, Sauter ER. 2004. Proteomic analysis to identify breast cancer biomarkers in nipple aspirate fluid. *Clin Cancer Res* **10:** (22) 7500.
Bahk YY, Kim SA, Kim JS, Euh HJ, Bai GH, Cho SN, Kim YS. 2004. Antigens secreted from *Mycobacterium tuberculosis*: Identification by proteomics approach and test for diagnostic marker. *Proteomics* **4:** (11) 3299.
Bergen HR, Zeldenrust SR, Butz ML, Snow DS, Dyck PJ, Dyck PJB, Klein CJ, O'Brien JF, Thibodeau SN, Muddiman DC. 2004. Identification of transthyretin variants by sequential proteomic and genomic analysis. *Clin Chem* **50:** (9) 1544.
Bosinger SE, Hosiawa KA, Cameron MJ, Persad D, Rang LS, Xu LL, Boulassel MR, Parenteau M, Fournier J, Rud EW *et al*. 2004. Gene expression profiling of host response in models of acute HIV infection. *J Immunol* **173:** (11) 6858.
Bull TM, Coldren CD, Moore M, Sotto-Santiago SM, Pham DV, Nana-Sinkam SP, Voelkel NF, Geraci MW. 2004. Gene microarray analysis of peripheral blood cells in pulmonary arterial hypertension. *Am J Respir Crit Care Med* **170:** (8) 911.
Cho JW, Kim JJ, Park SG, Lee DH, Lee SC, Kim HJ, Park BC, Cho SY. 2004. Identification of B-cell translocation gene 1 as a biomaker for monitoring the remission of acute myeloid leukemia. *Proteomics* **4:** (11) 3456.
Chung YW, Oh HY, Kim JY, Kim JH, Kim IY. 2004. Allergen-induced proteolytic cleavage of annexin-1 and activation of cytosolic phospholipase $A_2$ in the lungs of a mouse model of asthma. *Proteomics* **4:** (11) 3328.
De Fourmestraux V, Neubauer H, Poussin C, Farmer P, Falquet L, Burcelin R, Delorenzi M, Thorens B. 2004. Transcript profiling suggests that differential metabolic adaptation of mice to a high fat diet is associated with changes in liver to muscle lipid fluxes. *J Biol Chem* **279:** (49) 50743.
Desai KV, Michalowska AM, Kondaiah P, Ward JM, Shih JH, Green JE. 2004. Gene expression profiling identifies a unique androgen-mediated inflammatory/immune signature and a PTEN (phosphatase and tensin homolog deleted on chromosome 10)-mediated apoptotic response specific to the rat ventral prostate. *Mol Endocrinol* **18:** (12) 2895.
Ge Y, Rajkumar L, Guzman RC, Nandi S, Patton WF, Agnew BJ. 2004. Multiplexed fluorescence detection of phosphorylation, glycosylation, and total protein in the proteomic analysis of breast cancer refractoriness. *Proteomics* **4:** (11) 3464.
Gillanders EM, Xu JF, Chang BL, Lange EM, Wiklund F, Bailey-Wilson JE, Baffoe-Bonnie A, Jones M, Gildea D, Riedesel E *et al*. 2004. Combined genome-wide scan for prostate cancer susceptibility genes. *J Nat Cancer Inst* **96:** (16) 1240.
Grunblatt E, Mandel S, Jacob-Hirsch J, Zeligson S, Amariglo N, Rechavi G, Li J, Ravid R, Roggendorf W, Riederer P *et al*. 2004. Gene expression profiling of parkinsonian substantia nigra pars compacta: Alterations in ubiquitin-proteasome, heat shock protein, iron and oxidative stress regulated proteins, cell adhesion/cellular matrix and vesicle trafficking genes. *J Neural Transm* **111:** (12) 1543.
Horcajadas JA, Riesewijk A, Martin J, Cervero A, Mosselman S, Pellicer A, Simon C. 2004. Global gene expression profiling of human endometrial receptivity. *J Reprod Immunol* **63:** (1) 41.
Huang CM. 2004. Comparative proteomic analysis of human whole saliva. *Arch Oral Biol* **49:** (12) 951.
Ishido M, Masuo Y. 2004. Transcriptome of pituitary adenylate cyclase-activating polypeptide-differentiated PC12 cells. *Regul Pept* **123:** (1-3) 15.
Israeli O, Gotlieb WH, Friedman E, Korach J, Friedman E, Goldman B, Zeltser A, Ben Baruch G, Rienstein S, Aviram-Goldring A. 2004. Genomic analyses of primary and metastatic serous epithelial ovarian cancer. *Cancer Genet Cytogenet* **154:** (1) 16.
Jang JS, Cho HY, Lee YJ, Ha WS, Kim HW. 2004. The differential proteome profile of stomach cancer: Identification of the biomarker candidates. *Oncol Res* **14:** (10) 491.
Jang WG, Kim HS, Park KG, Park YB, Yoon KH, Han SW, Hur SH, Park KS, Lee IK. 2004. Analysis of proteome and transcriptome of tumor necrosis factor α stimulated vascular smooth muscle cells with or without α lipoic acid. *Proteomics* **4:** (11) 3383.
Jansen E, Laven JSE, Dommerholt HBR, Polman J, Van Rijt C, Van den Hurk C, Westland J, Mosselman S, Fauser BCJM. 2004. Abnormal gene expression profiles in human ovaries from polycystic ovary syndrome patients. *Mol Endocrinol* **18:** (12) 3050.
Katanasaka Y, Shimizu K, Naitou H, Ohashi N, Nakayama T, Oku N. 2004. Proteomic analysis of tumor angiogenic model by 2D-DIGE. *Yakugaku Zasshi* **124:** (S4) 309.
Khanna S, Cheng G, Gong B, Mustari MJ, Porter JD. 2004. Genome-wide transcriptional profiles are consistent with functional specialization of the extraocular muscle layers. *Invest Ophthalmol Vis Sci* **45:** (9) 3055.
Kim NK, Joh JH, Park HR, Kim OH, Park BY, Lee CS. 2004. Differential expression profiling of the proteomic and their mRNAs in porcine white and red skeletal muscles. *Proteomics* **4:** (11) 3422.
Konradi C, Westin JE, Carta M, Eaton ME, Kuter K, Dekundy A, Lundblad M, Cenci MA. 2004. Transcriptome analysis in a rat model of L-DOPA-induced dyskinesia. *Neurobiol Dis* **17:** (2) 219.
Kuerer HM, Coombes KR, Chen JN, Xiao LC, Clarke C, Fritsche H, Krishnamurthy S, Marcy S, Hung MC, Hunt KK. 2004. Association between ductal fluid proteomic expression profiles and the presence of lymph node metastases in women with breast cancer. *Surgery* **136:** (5) 1061.
Kutuzova GD, DeLuca HF. 2004. Gene expression profiles in rat intestine identify pathways for 1,25-dihydroxyvitamin $D_3$ stimulated calcium absorption and clarify its immunomodulatory properties. *Arch Biochem Biophys* **432:** (2) 152.
Lacayo NJ, Meshinchi S, Kinnunen P, Yu R, Wang Y, Stuber CM, Douglas L, Wahab R, Becton DL, Weinstein H *et al*. 2004. Gene expression profiles at diagnosis in *de novo* childhood AML patients identify FLT3 mutations with good clinical outcomes. *Blood* **104:** (9) 2646.
Lane CS, Nisar S, Griffiths WJ, Fuller BJ, Davidson BR, Hewes J, Welham KJ, Patterson LH. 2004. Identification of cytochrome P450 enzymes in human colorectal metastases and the surrounding liver: A proteomic approach. *Eur J Cancer* **40:** (14) 2127.
Lee HJ, Lee DY, Joo WA, Sul D, Lee E, Kim CW. 2004. Differential expression of proteins in rat plasma exposed to benzene. *Proteomics* **4:** (11) 3498.
Lee K, Kye M, Jang JS, Lee OJ, Kim T, Lim DB. 2004. Proteomic analysis revealed a strong association of a high level of α1-antitrypsin in gastric juice with gastric cancer. *Proteomics* **4:** (11) 3343.
Lee YW, Eum SY, Chen KC, Hennig B, Toborek M. 2004. Gene expression profile in interleukin-4-stimulated human vascular endothelial cells. *Mol Med* **10:** (1-6) 19.
Li LS, Kim H, Rhee H, Kim SH, Shin DH, Chung KY, Park KS, Paik YK, Chang J, Kim H. 2004. Proteomic analysis distinguishes basaloid carcinoma as a distinct subtype of nonsmall cell lung carcinoma. *Proteomics* **4:** (11) 3394.
Lia LJ, Cheng DM, Wang J, Duong DM, Losik TG, Gearing M, Rees HD, Lah JJ, Levey AI, Peng JM. 2004. Proteomic characterization of postmortem amyloid plaques isolated by laser capture microdissection. *J Biol Chem* **279:** (35) 37061.
Liu X, Mohamed JA, Ruan RS. 2004. Analysis of differential gene expression in the cochlea and kidney of mouse by cDNA microarrays. *Hear Res* **197:** (1-2) 35.
Midorikawa T, Chikazawa T, Yoshino T, Takada K, Arase S. 2004. Dif-

Copyright © 2005 John Wiley & Sons, Ltd. 

ferent gene expression profile observed in dermal papilla cells related to androgenic alopecia by DNA macroarray analysis. *J Dermatol Sci* **36:** (1) 25.
Mogass M, York TP, Li L, Rujirabanjerd S, Shiang R. 2004. Genomewide analysis of gene expression associated with Tcof1 in mouse neuroblastoma. *Biochem Biophys Res Commun* **325:** (1) 124.
Mohring T, Kellmann M, Jurgens M, Schrader M. 2005. Top-down identification of endogenous peptides up to 9 kDa in cerebrospinal fluid and brain tissue by nanoelectrospray quadrupole time-of-flight tandem mass spectrometry. *J Mass Spectrom* **40:** (2) 214.
Noh KS, Lee DY, Cha JH, Joo WA, Lee E, Kim CW. 2004. Protein biomarkers in the plasma of workers occupationally exposed to polycyclic aromatic hydrocarbons. *Proteomics* **4:** (11) 3505.
Norden AGW, Sharratt P, Cutillas PR, Cramer R, Gardner SC, Unwin RJ. 2004. Quantitative amino acid and proteomic analysis: Very low excretion of polypeptides >750 Da in normal urine. *Kidney Int* **66:** (5) 1994.
Oh J, Pyo JH, Jo EH, Hwang SI, Kang SC, Jung JH, Park EK, Kim SY, Choi JY, Lim J. 2004. Establishment of a near-standard two-dimensional human urine proteomic map. *Proteomics* **4:** (11) 3485.
Oh S, Im H, Oh E, Lee J, Khim JY, Mun J, Kim Y, Lee E, Kim J, Sul D. 2004. Effects of benzo(a)pyrene on protein expression in Jurkat T-cells. *Proteomics* **4:** (11) 3514.
Park B, Oh SH, Seong JK, Paik YK. 2004. A strain-specific alteration of proteomic expression in mouse liver fructose 1,6-bisphosphatase isoforms by alcohol. *Proteomics* **4:** (11) 3413.
Park B, Jeong SK, Lee WS, Seong JK, Paik YK. 2004. A simple pattern classification method for alcohol-responsive proteins that are differentially expressed in mouse brain. *Proteomics* **4:** (11) 3369.
Park YD, Kim SY, Jang HS, Seo EY, Namkung JH, Park HS, Cho SY, Paik YK, Yang JM. 2004. Towards a proteomic analysis of atopic dermatitis: A two-dimensional-polyacrylamide gel electrophoresis/mass spectrometric analysis of cultured patient-derived fibroblasts. *Proteomics* **4:** (11) 3446.
Parton LE, Diraison F, Neill SE, Ghosh SK, Rubino MA, Bisi JE, Briscoe CP, Rutter GA. 2004. Impact of PPARγ overexpression and activation on pancreatic islet gene expression profile analyzed with oligonucleotide microarrays. *Am J Physiol* **287:** (3) E390.
Paulson L, Persson R, Karlsson G, Silberring J, Bierczynska-Krzysik A, Ekman R, Westman-Brinkmalm A. 2005. Proteomics and peptidomics in neuroscience. Experience of capabilities and limitations in a neurochemical laboratory. *J Mass Spectrom* **40:** (2) 202.
Pisarchik A, Wortsman J, Slominski A. 2004. A novel microarray to evaluate stress-related genes in skin: Effect of ultraviolet light radiation. *Gene* **341:** 199.
Qian A, Meals RA, Rajfer J, Gonzalez-Cadavid NFG. 2004. Comparison of gene expression profiles between Peyronie's disease and Dupuytren's contracture. *Urology* **64:** (2) 399.
Quintana FJ, Hagedorn PH, Elizur G, Merbl Y, Domany E, Cohen IR. 2004. Functional immunomics: Microarray analysis of lgG autoantibody repertoires predicts the future response of mice to induce diabetes. *Proc Natl Acad Sci U S A* **101:** (Suppl 2) 14615.
Roelofsen H, Balgobind R, Vonk RJ. 2004. Proteomic analyzes of copper metabolism in an *in vitro* model of Wilson disease using surface enhanced laser desorption/ionization-time of flight-mass spectrometry. *J Cell Biochem* **93:** (4) 732.
Roh GS, Shin Y, Seo SW, Yoon BR, Yeo S, Park SJ, Cho JW, Kwack K. 2004. Proteome analysis of differential protein expression allergen-induced asthmatic mice lung after dexamethasone treatment. *Proteomics* **4:** (11) 3318.
Ross ME, Mahfouz R, Onciu M, Liu HC, Zhou XD, Song GC, Shurtleff SA, Pounds S, Cheng C, Ma J *et al*. 2004. Gene expression profiling of pediatric acute myelogenous leukemia. *Blood* **104:** (12) 3679.
Sabounchi-Schutt F, Mikko M, Eklund A, Grunewald J, Astrom J. 2004. Serum protein pattern in sarcoidosis analysed by a proteomics approach. *Sarcoidosis Vasc Diffuse Lung Dis* **21:** (3) 182.
Samimi G, Manorek G, Castel R, Breaux JK, Cheng T, Berry CC, Los G, Howell SB. 2005. cDNA microarray-based identification of genes and pathways associated with oxaliplatin resistance. *Cancer Chemother Pharmacol* **55:** (1) 1.
Shin SJ, Lee SE, Boo JH, Kim M, Yoon YD, Kim SI, Mook-Jung I. 2004. Profiling proteins related to amyloid deposited brain of Tg2576 mice. *Proteomics* **4:** (11) 3359.
Shipkova M, Spielbauer B, Voland A, Grone HJ, Armstrong VW, Oellerich M, Wieland E. 2004. cDNA microarray analysis reveals new candidate genes possibly linked to side effects under mycophenolate mofetil therapy. *Transplantation* **78:** (8) 1145.
Stentz FB, Kitabchi AE. 2004. Transcriptome and proteome expression in activated human CD4 and CD8 T-lymphocytes. *Biochem Biophys Res Commun* **324:** (2) 692.
Strom CC, Kruhoffer M, Knudsen S, Stensgaard-Hansen F, Jonassen TEN, Orntoft TF, Haunso S, Sheikh SP. 2004. Identification of a core set of genes that signifies pathways underlying cardiac hypertrophy. *Comp Funct Genom* **5:** (6-7) 459.
Suh SK, Hood BL, Kim BJ, Conrads TP, Veenstra TD, Song BJ. 2004. Identification of oxidized mitochondria proteins in alcohol-exposed human hepatoma cells and mouse liver. *Proteomics* **4:** (11) 3401.
Takada M, Tada M, Tamoto E, Kawakami A, Murakawa K, Shindoh G, Teramoto K, Matsunaga A, Komuro K, Kanai M *et al*. 2004. Prediction of lymph node metastasis by analysis of gene expression profiles in non-small cell lung cancer. *J Surg Res* **122:** (1) 61.
Tenedini E, Fagioli ME, Vianelli N, Tazzari PL, Ricci F, Tagliafico E, Ricci P, Gugliotta L, Martinelli G, Tura S *et al*. 2004. Gene expression profiling of normal and malignant CD34-derived megakaryocytic cells. *Blood* **104:** (10) 3126.
Toruner GA, Ulger C, Alkan M, Galante AT, Rinaggio J, Wilk R, Tian B, Soteropoulos P, Hameed MR, Schwalb MN *et al*. 2004. Association between gene expression profile and tumor invasion in oral squamous cell carcinoma. *Cancer Genet Cytogenet* **154:** (1) 27.
Trempat P, Villalva C, Xerri L, Armstrong F, Duplantier MM, Delsol G, Brousset P. 2004. Gene expression profiling in anaplastic large cell lymphoma and Hodgkin's disease. *Leuk Lymphoma* **45:** (10) 2001.
Tromp G, Kuivaniemi H, Romero R, Chaiworapongsa T, Kim YM, Kim MR, Maymon E, Edwin S. 2004. Genome-wide expression profiling of fetal membranes reveals a deficient expression of proteinase inhibitor 3 in premature rupture of membranes. *Am J Obstet Gynecol* **191:** (4) 1331.
Wood JR, Ho CKM, Nelson-Degrave VL, McAllister JM, Strauss JF. 2004. The molecular signature of polycystic ovary syndrome (PCOS) theca cells defined by gene expression profiling. *J Reprod Immunol* **63:** (1) 51.
Yan L, Ge H, Li H, Lieber SC, Natividad F, Resuello RRG, Kim SJ, Akeju S, Sun A, Loo K *et al*. 2004. Gender-specific proteomic alterations in glycolytic and mitochondrial pathways in aging monkey hearts. *J Mol Cell Cardiol* **37:** (5) 921.
Yeo M, Park HK, Kim DK, Cho SW, Kim YS, Cho SY, Paik YK, Hahm KB. 2004. Restoration of heat shock protein70 suppresses gastric mucosal inducible nitric oxide synthase expression induced by *Helicobacter pylori*. *Proteomics* **4:** (11) 3335.
Yeo S, Roh GS, Kim DH, Lee JM, Seo SW, Cho JW, Kim CW, Kwack K. 2004. Quantitative profiling of plasma peptides in asthmatic mice using liquid chromatography and mass spectrometry. *Proteomics* **4:** (11) 3308.
Yi R, He QY, Fan JQ, Jones B, Yuan Z, Yi X, Cheung CY, Wu A, Chiu JF, Peiris JSM *et al*. 2004. The use of proteomics in the discovery of serum biomarkers from patients with severe acute respiratory syndrome. *Proteomics* **4:** (11) 3477.
Yuan JP, Li T, Chen HB, Li ZH, Yang GZ, Hu BY, Shi XD, Tong SQ, Li YX, Guo XK. 2004. Analysis of gene expression profile in gastric cancer cells stimulated with *Helicobacter pylori* isogenic strains. *J Med Microbiol* **53:** (10) 965.
Yuan X, Desiderio DM. 2005. Human cerebrospinal fluid peptidomics. *J Mass Spectrom* **40:** (2) 176.

### 8 EST, cDNA and other clone resources

Ahmed F, Torrado M, Zinovieva RD, Senatorov VV, Wistow G, Tomarev SI. 2004. Gene expression profile of the rat eye iridocorneal angle: NEIBank expressed sequence tag analysis. *Invest Ophthalmol Vis Sci* **45:** (9) 3081.
Chen YR, Lee YR, Wang SY, Chang ST, Shaw JF, Chu FH. 2004. Establishment of expressed sequence tags from taiwania (*Taiwania cryptomerioides* Hayata) seedling cDNA. *Plant Sci* **167:** (4) 955.
Ebbole DJ, Jin Y, Thon M, Pan HQ, Bhattarai E, Thomas T, Dean R. 2004. Gene discovery and gene expression in the rice blast fungus, *Magnaporthe grisea*: Analysis of expressed sequence tags. *Mol Plant Microbe Interact* **17:** (12) 1337.
Hossain KG, Kalavacharla V, Lazo GR, Hegstad J, Wentz MJ, Kianian PMA, Simons K, Gehlhar S, Rust JL, Syamala RR *et al*. 2004. A chromosome bin map of 2148 expressed sequence tag loci of wheat

Copyright © 2005 John Wiley & Sons, Ltd. 

homoeologous group 7. *Genetics* **168:** (2) 687.
Jiang H, Whitworth KM, Bivens NJ, Ries JE, Woods RJ, Forrester LJ, Springer GK, Mathialagan N, Agca C, Prather RS *et al*. 2004. Large-scale generation and analysis of expressed sequence tags from porcine ovary. *Biol Reprod* **71:** (6) 1991.
Lazo GR, Chao S, Hummel DD, Edwards H, Crossman CC, Lui N, Matthews DE, Carollo VL, Hane DL, You FM *et al*. 2004. Development of an expressed sequence tag (EST) resource for wheat (*Triticum aestivum* L.): EST generation, unigene analysis, probe selection and bioinformatics for a 16,000-locus bin-delineated map. *Genetics* **168:** (2) 585.
Miftahudin, Ross K, Ma XF, Mahmoud AA, Layton J, Milla MAR, Chikmawati T, Ramalingam J, Feril O, Pathan MS *et al*. 2004. Analysis of expressed sequence tag loci on wheat chromosome group 4. *Genetics* **168:** (2) 651.
Peng JH, Zadeh H, Lazo GR, Gustafson JP, Chao S, Anderson OD, Qi LL, Echalier B, Gill BS, Dilbirligi M *et al*. 2004. Chromosome bin map of expressed sequence tags in homoeologous group 1 of hexaploid wheat and homoeology with rice and *Arabidopsis*. *Genetics* **168:** (2) 609.
Pinto LR, Oliveira KM, Ulian EC, Garcia AAF, De Souza AP. 2004. Survey in the sugarcane expressed sequence tag database (SUCEST) for simple sequence repeats. *Genome* **47:** (5) 795.
Qi LL, Echalier B, Chao S, Lazo GR, Butler GE, Anderson OD, Akhunov ED, Dvorak J, Linkiewicz AM, Ratnasiri A *et al*. 2004. A chromosome bin map of 16,000 expressed sequence tag loci and distribution of genes among the three genomes of polyploid wheat. *Genetics* **168:** (2) 701.
Randhawa HS, Dilbirligi M, Sidhu D, Erayman M, Sandhu D, Bondareva S, Chao S, Lazo GR, Anderson OD, Miftahudin *et al*. 2004. Deletion mapping of homoeologous group 6-specific wheat expressed sequence tags. *Genetics* **168:** (2) 677.
Rishi AS, Munir S, Kapur V, Nelson ND, Goyal A. 2004. Identification and analysis of safener-inducible expressed sequence tags in *Populus* using a cDNA microarray. *Planta* **220:** (2) 296.
Wilson ID, Barker GLA, Beswick RW, Shepherd SK, Lu CG, Coghill JA, Edwards D, Owen P, Lyons R, Parker JS *et al*. 2004. A transcriptomics resource for wheat functional genomics. *Plant Biotechnol J* **2:** (6) 495.
Yu JK, Dake TM, Singh S, Benscher D, Li WL, Gill B, Sorrells ME. 2004. Development and mapping of EST-derived simple sequence repeat markers for hexaploid wheat. *Genome* **47:** (5) 805.
Zhang D, Choi DW, Wanamaker S, Fenton RD, Chin A, Malatrasi M, Turuspekov Y, Walia H, Akhunov ED, Kianian P, et al. 2004. Construction and evaluation of cDNA libraries for large-scale expressed sequence tag sequencing in wheat (*Triticum aestivum* L.). *Genetics* **168:** (2) 595.

### 9 Functional genomics

Clark AT, Goldowitz D, Takahashi JS, Vitaterna MH, Siepka SM, Peters LL, Frankel WN, Carlson GA, Rossant J, Nadeau JH *et al*. 2004. Implementing large-scale ENU mutagenesis screens in North America. *Genetica* **122:** (1) 51.
Kumar A, Seringhaus M, Biery MC, Sarnovsky RJ, Umansky L, Piccirillo S, Heidtman M, Cheung KH, Dobry CJ, Gerstein MB *et al*. 2004. Large-scale mutagenesis of the yeast genome using a Tn7-derived multipurpose transposon. *Genome Res* **14:** (10A) 1975.
Leple JC, Dejardin A, Laurans F, Pilate G, Goue N, Label P, Beritognolo I, Boizot N, Breton C. 2004. Physiology and genomics of wood development (French). *Biofutur* **247:** 43.
Luscombe NM, Babu MM, Yu HY, Snyder M, Teichmann SA, Gerstein M. 2004. Genomic analysis of regulatory network dynamics reveals large topological changes. *Nature* **431:** (7006) 308.
Martin F, Kohler A. 2004. Structural and functional genomics of the poplar (French). *Biofutur* **247:** 38.
Pan XW, Yuan DS, Xiang D, Wang XL, Sookhai-Mahadeo S, Bader JS, Hieter P, Spencer F, Boeke JD. 2004. A robust toolkit for functional profiling of the yeast genome. *Mol Cell* **16:** (3) 487.
Song WJ, Oin QW, Qiu J, Huang CH, Wang F, Hew CL. 2004. Functional genomics analysis of Singapore grouper iridovirus: Complete sequence determination and proteomic analysis. *J Virol* **78:** (22) 12576.

### I0 Transcriptomics

Braatsch S, Moskvin OV, Klug G, Gomelsky M. 2004. Responses of the *Rhodobacter sphaeroides* transcriptome to blue light under semiaerobic conditions. *J Bacteriol* **186:** (22) 7726.
Contento AL, Kim SJ, Bassham DC. 2004. Transcriptome profiling of the response of *Arabidopsis* suspension culture cells to Suc starvation. *Plant Physiol* **135:** (4) 2330.
Gao HC, Wang Y, Liu XD, Yan TF, Wu LY, Alm E, Arkin A, Thompson DK, Zhou JZ. 2004. Global transcriptome analysis of the heat shock response of *Shewanella oneidensis*. *J Bacteriol* **186:** (22) 7796.
Gu WS, Bertone AL. 2004. Generation and performance of an equine-specific large-scale gene expression microarray. *Am J Vet Res* **65:** (12) 1664.
Han YP, Zhou DS, Pang X, Song YJ, Zhang L, Bao JY, Tong ZZ, Wang J, Guo ZB, Zhai JH *et al*. 2004. Microarray analysis of temperature-induced transcriptome of *Yersinia pestis*. *Microbiol Immunol* **48:** (11) 791.
Kirst M, Myburg AA, De Leon JPG, Kirst ME, Scott J, Sederoff R. 2004. Coordinated genetic regulation of growth and lignin revealed by quantitative trait locus analysis of cDNA microarray data in an interspecific backcross of eucalyptus. *Plant Physiol* **135:** (4) 2368.
Phadtare S, Inouye M. 2004. Genome-wide transcriptional analysis of the cold shock response in wild-type and cold-sensitive, quadruple-csp-deletion strains of *Escherichia coli*. *J Bacteriol* **186:** (20) 7007.
Verhagen BWM, Glazebrook J, Zhu T, Chang HS, Van Loon LC, Pieterse CMJ. 2004. The transcriptome of rhizobacteria-induced systemic resistance in *Arabidopsis*. *Mol Plant Microbe Interact* **17:** (8) 895.
Yamakawa S, Ando K, Chisaka A, Yoshida K, Shinmyo A, Kohchi T. 2004. Systematic transient assays of promoter activities for leaf-specific genes identified by gene-expression profiling with cDNA microarrays in *Arabidopsis thaliana*. *J Biosci Bioeng* **98:** (2) 140.
Yamashita T, Honda M, Takotori H, Nishino R, Hoshino N, Kaneko S. 2004. Genome-wide transcriptome mapping analysis identifies organ-specific gene expression patterns along human chromosomes. *Genomics* **84:** (5) 867.

### II Proteomics

Aivaliotis M, Corvey C, Tsirogianni I, Karas M, Tsiotis G. 2004. Membrane proteome analysis of the green-sulfur bacterium *Chlorobium tepidum*. *Electrophoresis* **25:** (20) 3468.
Alexandersson E, Saalbach G, Larsson C, Kjellbom P. 2004. *Arabidopsis* plasma membrane proteomics identifies components of transport, signal transduction and membrane trafficking. *Plant Cell Physiol* **45:** (11) 1543.
Bae TJ, Kim MS, Kim JW, Kim BW, Choo HJ, Lee JW, Kim KB, Lee CS, Kim JH, Chang SY *et al*. 2004. Lipid raft proteome reveals ATP synthase complex in the cell surface. *Proteomics* **4:** (11) 3536.
Bonchev D. 2004. Complexity analysis of yeast proteome network. *Chem Biodivers* **1:** (2) 312.
Che FY, Fricker LD. 2005. Quantitative peptidomics of mouse pituitary: Comparison of different stable isotopic tags. *J Mass Spectrom* **40:** (2) 238.
Colditz F, Nyamsuren O, Niehaus K, Eubel H, Braun HP, Krajinski F. 2004. Proteomic approach: Identification of *Medicago truncatula* proteins induced in roots after infection with the pathogenic oomycete *Aphanomyces euteiches*. *Plant Mol Biol* **55:** (1) 109.
Kang JG, Pyo YJ, Cho JW, Cho MH. 2004. Comparative proteome analysis of differentially expressed proteins induced by $K^+$ deficiency in *Arabidopsis thaliana*. *Proteomics* **4:** (11) 3549.
Kao WC, Chen YR, Yi EC, Lee H, Tian Q, Wu KM, Tsai SF, Yu SSF, Chen YJ, Aebersold R *et al*. 2004. Quantitative proteomic analysis of metabolic regulation by copper ions in *Methylococcus capsulatus* (Bath). *J Biol Chem* **279:** (49) 51554.
Kim HJ, Lee DY, Lee DH, Park YC, Kweon DH, Ryu YW, Seo JH. 2004. Strategic proteome analysis of *Candida magnoliae* with an unsequenced genome. *Proteomics* **4:** (11) 3588.
Kim KB, Kim SI, Choo HJ, Kim JH, Ko YG. 2004. Two-dimensional electrophoretic analysis reveals that lipid rafts are intact at physiological temperature. *Proteomics* **4:** (11) 3527.
Kim ST, Kim SG, Hwang DH, Kang SY, Kim HJ, Lee BH, Lee JJ, Kang KY. 2004. Proteomic analysis of pathogen-responsive proteins

Copyright © 2005 John Wiley & Sons, Ltd.

from rice leaves induced by rice blast fungus, *Magnaporthe grisea*. *Proteomics* **4:** (11) 3569.
Kim ST, Yu S, Kim SG, Kim HJ, Kang SY, Hwang DH, Jang YS, Kang KY. 2004. Proteome analysis of rice blast fungus (*Magnaporthe grisea*) proteome during appressorium formation. *Proteomics* **4:** (11) 3579.
Kim YS, Hwang SY, Oh S, Sohn H, Kang HY, Lee JH, Cho EW, Kim JY, Yoo JS, Kim NS *et al*. 2004. Identification of target proteins of *N*-acetylglucosaminyl-transferase V and fucosyltransferase 8 in human gastric tissues by glycomic approach. *Proteomics* **4:** (11) 3353.
Kurogochi M, Nishimura SI, Lee YC. 2004. Mechanism-based fluorescent labeling of β-galactosidases - An efficient method in proteomics for glycoside hydrolases. *J Biol Chem* **279:** (43) 44704.
Lee JS, Hong US, Lee TH, Yoon SK, Yoon JB. 2004. Mass spectrometric analysis of tumor necroses factor receptor-associated factor 1 ubiquitination mediated by cellular inhibitor of apoptosis 2. *Proteomics* **4:** (11) 3376.
Lee MS, Joo WA, Kim CW. 2004. Identification of a novel protein D3UPCA from *Halobacterium salinarum* and prediction of its function. *Proteomics* **4:** (11) 3622.
Park HJ, Lee DH, Park SG, Lee SC, Cho S, Kim HK, Kim JJ, Bael H, Park BC. 2004. Proteome analysis of red deer antlers. *Proteomics* **4:** (11) 3642.
Park SJ, Joo WA, Choi J, Lee SH, Kim CW. 2004. Identification and characterization of inosine monophosphate dehydrogenase from *Halobacterium salinarum*. *Proteomics* **4:** (11) 3632.
Peltier JB, Ytterberg AJ, Sun Q, Van Wijk KJ. 2004. New functions of the thylakoid membrane proteome of *Arabidopsis thaliana* revealed by a simple, fast, and versatile fractionation strategy. *J Biol Chem* **279:** (47) 49367.
Phee BK, Cho JH, Park S, Jung JH, Lee YH, Jeon JS, Bhoo SH, Hahn TR. 2004. Proteomic analyses of the response of *Arabidopsis* chloroplast proteins to high light stress. *Proteomics* **4:** (11) 3560.
Ritter B, Blondeau F, Denisov AY, Gehring K, McPherson PS. 2004. Molecular mechanisms in clathrin-mediated membrane budding revealed through subcellular proteomics. *Biochem Soc Trans* **32:** (5) 769.
Schiltz S, Gallardo K, Huart M, Negroni L, Sommerer N, Burstin J. 2004. Proteome reference maps of vegetative tissues in pea. An investigation of nitrogen mobilization from leaves during seed filling. *Plant Physiol* **135:** (4) 2241.
Seo JB, Kim HS, Jung GY, Nam MH, Chung JH, Kim JY, Jong SY, Kim CW, Kwon O. 2004. Psychrophilicity of *Bacillus psychrosaccharolyticus*: A proteomic study. *Proteomics* **4:** (11) 3654.
Shin YS, Lee EG, Shin GW, Kim YR, Lee EY, Kim JH, Jang H, Gershwin LJ, Kim DY, Kim YH, et al. 2004. Identification of antigenic proteins from *Neospora caninum* recognized by bovine immunoglobulins M, E, A and G using immunoproteomics. *Proteomics* **4:** (11) 3600.
Smales CM, Dinnis DM, Stansfield SH, Alete D, Sage EA, Birch JR, Racher AJ, Marshall CT, James DC. 2004. Comparative proteomic analysis of GS-NSO murine myeloma cell lines with varying recombinant monoclonal antibody production rate. *Biotechnol Bioeng* **88:** (4) 474.
Tao LD, Yu XL, Snyder AP, Li L. 2004. Bacterial identification by protein mass mapping combined with an experimentally derived protein mass database. *Anal Chem* **76:** (22) 6609.
Utsubo R, Sonoda Y, Takahashi R, Iijima S, Aizu-Yokota E, Kasahara T. 2004. Proteome analysis of focal adhesion kinase (FAK)-overexpressing cells. *Biol Pharm Bull* **27:** (11) 1735.

### I2 Protein structural genomics

Page R, Stevens RC. 2004. Crystallization data mining in structural genomics: Using positive and negative results to optimize protein crystallization screens. *Methods* **34:** (3) 373.
Randall AZ, Baldi P, Villarreal LP. 2004. Structural proteomics of the poxvirus family. *Artif Intell Med* **31:** (2) 105.
Symersky J, Zhang Y, Schormann N, Li S, Bunzel R, Pruett P, Luan CH, Luo M. 2004. Structural genomics of *Caenorhabditis elegans*: Structure of the BAG domain. *Acta Crystallogr D Biol Crystallogr* **60:** (9) 1606.
Tempel W, Liu ZJ, Schubot FD, Shah A, Weinberg MV, Jenney FE, Arendall WB, Adams MWW, Richardson JS, Richardson DC *et al*. 2004. Structural genomics of *Pyrococcus furiosus*: X-ray crystallography reveals 3D domain swapping in rubrerythrin. *Proteins* **57:** (4) 878.

### I3 Metabolomics

Akesson M, Forster J, Nielsen J. 2004. Integration of gene expression data into genome-scale metabolic models. *Metab Eng* **6:** (4) 285.
Kim SI, Kim JY, Yun SH, Kim JH, Leem SH, Lee C. 2004. Proteome analysis of *Pseudomonas sp* K82 biodegradation pathways. *Proteomics* **4:** (11) 3610.
Lee AY, Park LC, Jang M, Cho S, Lee DH, Lee SC, Myung PK, Park SG. 2004. Identification of caspase-3 degradome by two-dimensional gel electrophoresis and matrix-assisted laser desorption/ionization-time of flight analysis. *Proteomics* **4:** (11) 3429.
Lee AY, Park SG, Kho CW, Park SY, Cho S, Lee SC, Lee DH, Myung PK, Park BC. 2004. Identification of the degradome of Isp-1, a major intracellular serine protease of *Bacillus subtilis*, by two-dimensional gel electrophoresis and matrix-assisted laser desorption/ionization-time of flight analysis. *Proteomics* **4:** (11) 3437.
Matsumoto F, Obayashi T, Sasaki-Sekimoto Y, Ohta H, Takamiya K, Masuda T. 2004. Gene expression profiling of the tetrapyrrole metabolic pathway in *Arabidopsis* with a mini-array system. *Plant Physiol* **135:** (4) 2379.

### I4 Genomic approaches to development

Curwen RS, Ashton PD, Johnston DA, Wilson RA. 2004. The *Schistosoma mansoni* soluble proteome: A comparison across four life-cycle stages. *Mol Biochem Parasitol* **138:** (1) 57.
Espinosa-Soto C, Padilla-Longoria P, Alvarez-Buylla ER. 2004. A gene regulatory network model for cell-fate determination during *Arabidopsis thaliana* flower development that is robust and recovers experimental gene expression profiles. *Plant Cell* **16:** (11) 2923.
Hung SC, Chang CF, Ma HL, Chen TH, Ho LLT. 2004. Gene expression profiles of early adipogenesis in human mesenchymal stem cells. *Gene* **340:** (1) 141.
Kearney JB, Wheeler SR, Estes P, Parente B, Crews ST. 2004. Gene expression profiling of the developing *Drosophila* CNS midline cells. *Dev Biol* **275:** (2) 473.
Kouchi H, Shimomura K, Hata S, Hirota A, Wu GJ, Kumagai H, Tajima S, Suganuma N, Suzuki A, Aoki T *et al*. 2004. Large-scale analysis of gene expression profiles during early stages of root nodule formation in a model legume, *Lotus japonicus*. *DNA Res* **11:** (4) 263.
Le Roch KG, Johnson JR, Florens L, Zhou YY, Santrosyan A, Grainger M, Yan SF, Williamson KC, Holder AA, Carucci DJ *et al*. 2004. Global analysis of transcript and protein levels across the *Plasmodium falciparum* life cycle. *Genome Res* **14:** (11) 2308.
Lee RD, Rhee GS, An SM, Kim SS, Kwack SJ, Seok JH, Chae SY, Park CH, Yoon HJ, Cho DH *et al*. 2004. Differential gene profiles in developing embryo and fetus after *in utero* exposure to ethanol. *J Toxicol Environ Health A* **67:** (23-24) 2073.
Linney E, Dobbs-McAuliffe B, Sajadi H, Malek RL. 2004. Microarray gene expression profiling during the segmentation phase of zebrafish development. *Comp Biochem Physiol C Pharmacol Toxicol* **138:** (3) 351.
Mirkin S, Nikas G, Hsiu JG, Diaz J, Oehninger S. 2004. Gene expression profiles and structural/functional features of the peri-implantation endometrium in natural and gonadotropin-stimulated cycles. *J Clin Endocrinol Metab* **89:** (11) 5742.
Omoto CK, Toso M, Tang KL, Sibley LD. 2004. Expressed sequence tag (EST) analysis of Gregarine gametocyst development. *Int J Parasitol* **34:** (11) 1265.
Rao RR, Calhoun JD, Qin XT, Rekaya R, Clark JK, Stice SL. 2004. Comparative transcriptional profiling of two human embryonic stem cell lines. *Biotechnol Bioeng* **88:** (3) 273.
Tao W, Wang M, Voss ED, Cocklin RR, Smith JA, Cooper SH, Broxmeyer HE. 2004. Comparative proteomic analysis of human $CD34^+$ stem/progenitor cells and mature $CD15^+$ myeloid cells. *Stem Cells* **22:** (6) 1003.
Usui T, Yamagami S, Yokoo S, Mimura T, Ono K, Amano S. 2004. Gene expression profile in corneal neovascularization identified by immunology related macroarray. *Mol Vis* **10:** (99-101) 832.
Wang ML, Hsu CM, Chang LC, Wang CS, Su TH, Huang YJJ, Jiang LW, Jauh GY. 2004. Gene expression profiles of cold-stored and fresh pollen to investigate pollen germination and growth. *Plant Cell Physiol* **45:** (10) 1519.

Copyright © 2005 John Wiley & Sons, Ltd.

### I5 Technological advances

Chan EWS, Chattopadhaya S, Panicker RC, Huang X, Yao SQ. 2004. Developing photoactive affinity probes for proteomic profiling: Hydroxamate-based probes for metalloproteases. *J Am Chem Soc* **126:** (44) 14435.

Dafforn A, Chen P, Deng G, Herrler M, Iglehart D, Koritala S, Lato S, Pillarisetty S, Purohit R, Wang M *et al*. 2004. Linear mRNA amplification from as little as 5 ng total RNA for global gene expression analysis. *Biotechniques* **37:** (5) 854.

Inza I, Larranaga P, Blanco R, Cerrolaza AJ. 2004. Filter *versus* wrapper gene selection approaches in DNA microarray domains. *Artif Intell Med* **31:** (2) 91.

Kuhn K, Baker SC, Chudin E, Lieu MH, Oeser S, Bennett H, Rigault P, Barker D, McDaniel TK, Chee MS. 2004. A novel, high-performance random array platform for quantitative gene expression profiling. *Genome Res* **14:** (11) 2347.

Li KC, Liu CT, Sun W, Yuan SS, Yu TW. 2004. A system for enhancing genome-wide coexpression dynamics study. *Proc Natl Acad Sci U S A* **101:** (44) 15561.

Pedrioli PGA, Eng JK, Hubley R, Vogelzang M, Deutsch EW, Raught B, Pratt B, Nilsson E, Angeletti RH, Apweiler R *et al*. 2004. A common open representation of mass spectrometry data and its application to proteomics research. *Nat Biotechnol* **22:** (11) 1459.

Ruotolo BT, Gillig KJ, Woods AS, Egan TF, Ugarov MV, Schultz JA, Russell DH. 2004. Analysis of phosphorylated peptides by ion mobility-mass spectrometry. *Anal Chem* **76:** (22) 6727.

Sieber SA, Mondala TS, Head SR, Cravatt BF. 2004. Microarray platform for profiling enzyme activities in complex proteomes. *J Am Chem Soc* **126:** (48) 15640.

Storms HF, Van der Heijden R, Tjaden UR, Van der Greef J. 2004. Capillary isoelectric focusing-mass spectrometry for shotgun approach in proteomics. *Electrophoresis* **25:** (20) 3461.

Stoyanova R, Upson JJ, Patriotis C, Ross EA, Henske EP, Datta K, Boman B, Clapper ML, Knudson AG, Bellacosa A. 2004. Use of RNA amplification in the optimal characterization of global gene expression using cDNA microarrays. *J Cell Physiol* **201:** (3) 359.

Van den Berg N, Crampton B, Hein I, Birch P, Berger D. 2004. High-throughput screening of suppression subtractive hybridization cDNA libraries using DNA microarray analysis. *Biotechniques* **37:** (5) 818.

Yoo C, Cooper GF. 2004. An evaluation of a system that recommends microarray experiments to perform to discover gene-regulation pathways. *Artif Intell Med* **31:** (2) 169.

Yuk JS, Jung SH, Jung JW, Hong DG, Han JA, Kim YM, Ha KS. 2004. Analysis of protein interactions on protein arrays by a wavelength interrogation-based surface plasmon resonance biosensor. *Proteomics* **4:** (11) 3468.

Zappacosta F, Annan RS. 2004. N-terminal isotope tagging strategy for quantitative proteomics: Results-driven analysis of protein abundance changes. *Anal Chem* **76:** (22) 6618.

### I6 Bioinformatics

Aburatani S, Goto K, Saito S, Fumoto M, Imaizumi A, Sugaya N, Murakami H, Sat M, Toh H, Horimoto K. 2004. ASIAN: A website for network inference. *Bioinformatics* **20:** (16) 2853.

Adryan B, Schuh R. 2004. Gene-Ontology-based clustering of gene expression data. *Bioinformatics* **20:** (16) 2851.

Alexandridis R, Lin SL, Irwin M. 2004. Class discovery and classification of tumor samples using mixture modeling of gene expression data - A unified approach. *Bioinformatics* **20:** (16) 2545.

Almeida LGP, Paixao R, Souza RC, Da Costa GC, Barrientos FJA, Dos Santos MT, De Almeida DF, Vasconcelos ATR. 2004. A System for Automated Bacterial (genome) Integrated Annotation - SABIA. *Bioinformatics* **20:** (16) 2832.

Attoor S, Dougherty ER, Chen Y, Bittner ML, Trent JM. 2004. Which is better for cDNA-microarray-based classification: Ratios or direct intensities. *Bioinformatics* **20:** (16) 2513.

Bajic VB, Tan SL, Suzuki Y, Sugano S. 2004. Promoter prediction analysis on the whole human genome. *Nat Biotechnol* **22:** (11) 1467.

Bidaut G, Ochs MF. 2004. ClutrFree: Cluster tree visualization and interpretation. *Bioinformatics* **20:** (16) 2869.

Boehm AM, Grosse-Coosmann F, Sickmann A. 2004. Command line tool for calculating theoretical MS spectra for given sequences. *Bioinformatics* **20:** (16) 2889.

Boutros PC, Okey AB. 2004. PUNS: Transcriptomic- and genomic-*in silico* PCR for enhanced primer design. *Bioinformatics* **20:** (15) 2399.

Bozdech Z, Ginsburg H. 2004. Antioxidant defense in *Plasmodium falciparum* - Data mining of the transcriptome. *Malaria J* **3:** 23.

Broet P, Lewin A, Richardson S, Dalmasso C, Magdelenat H. 2004. A mixture model-based strategy for selecting sets of genes in multiclass response microarray experiments. *Bioinformatics* **20:** (16) 2562.

Casstevens TM, Buckler ES. 2004. GDPC: Connecting researchers with multiple integrated data sources. *Bioinformatics* **20:** (16) 2839.

Chalk AM, Wennerberg M, Sonnhammer ELL. 2004. Sfixem - Graphical sequence feature display in Java. *Bioinformatics* **20:** (15) 2488.

Chen HC, Lee HC, Lin TY, Li WH, Chen BS. 2004. Quantitative characterization of the transcriptional regulatory network in the yeast cell cycle. *Bioinformatics* **20:** (12) 1914.

Chen L, Oughtred L, Berman HM, Westbrook J. 2004. TargetDB: A target registration database for structural genomics projects. *Bioinformatics* **20:** (16) 2860.

Chikayama E, Kurotani A, Kuroda Y, Yokoyama S. 2004. ProteoMix: An integrated and flexible system for interactively analyzing large numbers of protein sequences. *Bioinformatics* **20:** (16) 2836.

Choudhuri JV, Schleiermacher C, Kurtz S, Giegerich R. 2004. GenAlyzer: Interactive visualization of sequence similarities between entire genomes. *Bioinformatics* **20:** (12) 1964.

Csuros M, Milosavljevic A. 2004. Pooled Genomic Indexing (PGI): Analysis and design of experiments. *J Comput Biol* **11:** (5) 1001.

Dodd LE, Korn EL, McShane LM, Chandramouli GVR, Chuang EY. 2004. Correcting log ratios for signal saturation in cDNA microarrays. *Bioinformatics* **20:** (16) 2685.

Eriksson L, Antti H, Gottfries J, Holmes E, Johansson E, Lindgren F, Long I, Lundstedt T, Trygg J, Wold S. 2004. Using chemometrics for navigating in the large data sets of genomics, proteomics, and metabonomics (gpm). *Anal Bioanal Chem* **380:** (3) 419.

Flanagan K, Stevens R, Pocock M, Lee P, Wipat A. 2004. Ontology for genome comparison and genomic rearrangements. *Comp Funct Genom* **5:** (6-7) 537.

Fofanov Y, Luo Y, Katili C, Wang J, Belosludtsev Y, Powdrill T, Belapurkar C, Fofanov V, Li TB, Chumakov S *et al*. 2004. How independent are the appearances of *n*-mers in different genomes? *Bioinformatics* **20:** (15) 2421.

Friedman R, Ekollu V, Rose J, Hughes A. 2004. Dblox: a genome- wide test for ancient segmental duplication. *Bioinformatics* **20:** (16) 2834.

Fu Y, Yang Q, Sun RX, Li DQ, Zeng R, Ling CX, Gao W. 2004. Exploiting the kernel trick to correlate fragment ions for peptide identification *via* tandem mass spectrometry. *Bioinformatics* **20:** (12) 1948.

Gkoutos GV, Green ECJ, Mallon AM, Blake A, Greenaway S, Hancock JM, Davidson D. 2004. Ontologies for the description of mouse phenotypes. *Comp Funct Genom* **5:** (6-7) 545.

Gottfries J, Sjogren M, Holmberg B, Rosengren L, Davidsson P, Blennow K. 2004. Proteomics for drug target discovery. *Chemometr Intell Lab Syst* **73:** (1) 47.

Grad YH, Roth FP, Halfon MS, Church GM. 2004. Prediction of similarly acting *cis*-regulatory modules by subsequence profiling and comparative genomics in *Drosophila melanogaster* and *D. pseudoobscura*. *Bioinformatics* **20:** (16) 2738.

Gupta S, Zink D, Korn B, Vingron M, Haas SA. 2004. Genome wide identification and classification of alternative splicing based on EST data. *Bioinformatics* **20:** (16) 2579.

Gutierrez RA, Larson MD, Wilkerson C. 2004. The plant-specific database. Classification of *Arabidopsis* proteins based on their phylogenetic profile. *Plant Physiol* **135:** (4) 1888.

Higa RH, Montagner AJ, Towaga RC, Kuser PR, Yamagishi MEB, Mancini AL, Pappas G, Miura RT, Horita LG, Neshich G. 2004. ConSSeq: A web-based application for analysis of amino acid conservation based on HSSP database and within context of structure. *Bioinformatics* **20:** (12) 1983.

Hsu SYT. 2004. Bioinformatics in reproductive biology - Functional annotation based on comparative sequence analysis. *J Reprod Immunol* **63:** (1) 75.

Huang XQ, Ye L, Chou HH, Yang IH, Chao KM. 2004. Efficient combination of multiple word models for improved sequence comparison. *Bioinformatics* **20:** (16) 2529.

Ji LP, Tan KL. 2004. Mining gene expression data for positive and negative co-regulated gene clusters. *Bioinformatics* **20:** (16) 2711.

Keith JM, Adams P, Bryant D, Cochran DAE, Lala GH, Mitchelson KR.

Copyright © 2005 John Wiley & Sons, Ltd.

2004. Algorithms for sequence analysis *via* mutagenesis. *Bioinformatics* **20:** (15) 2401.

Korotkiy M, Middelburg R, Dekker H, Van Harmelen F, Lankelma J. 2004. A tool for gene expression based PubMed search through combining data sources. *Bioinformatics* **20:** (12) 1980.

Krishnan A, Tang F. 2004. Exhaustive whole-genome tandem repeats search. *Bioinformatics* **20:** (16) 2702.

Kumar A, Smith B, Novotny DD. 2004. Biomedical informatics and granularity. *Comp Funct Genom* **5:** (6-7) 501.

Kyoda K, Baba K, Onami S, Kitano H. 2004. DBRF-MEGN method: An algorithm for deducing minimum equivalent gene networks from large-scale gene expression profiles of gene deletion mutants. *Bioinformatics* **20:** (16) 2662.

Lanckriet GRG, De Bie T, Cristianini N, Jordan MI, Noble WS. 2004. A statistical framework for genomic data fusion. *Bioinformatics* **20:** (16) 2626.

Laval G, Excoffier L. 2004. SIMCOAL 2.0: A program to simulate genomic diversity over large recombining regions in a subdivided population with a complex history. *Bioinformatics* **20:** (15) 2485.

Lawrence CJ, Zmasek CM, Dawe RK, Malmberg RL. 2004. LumberJack: A heuristic tool for sequence alignment exploration and phylogenetic inference. *Bioinformatics* **20:** (12) 1977.

Lazzarato F, Franceschinis G, Botta M, Cordero F, Calogero RA. 2004. RRE: A tool for the extraction of non-coding regions surrounding annotated genes from genomic datasets. *Bioinformatics* **20:** (16) 2848.

Li S, Chou HH. 2004. LUCY2: An interactive DNA sequence quality trimming and vector removal tool. *Bioinformatics* **20:** (16) 2865.

Li T, Zhang CL, Ogihara M. 2004. A comparative study of feature selection and multiclass classification methods for tissue classification based on gene expression. *Bioinformatics* **20:** (15) 2429.

Liao JG, Lin Y, Selvanayagam ZE, Shih WCJ. 2004. A mixture model for estimating the local false discovery rate in DNA microarray analysis. *Bioinformatics* **20:** (16) 2694.

Liebel U, Kindler B, Pepperkok R. 2004. 'Harvester': A fast meta search engine of human protein resources. *Bioinformatics* **20:** (12) 1962.

Luo F, Khan L, Bastani F, Yen IL, Zhou JZ. 2004. A dynamically growing self-organising tree (DGSOT) for hierarchical clustering gene expression profiles. *Bioinformatics* **20:** (16) 2605.

Luscombe NM, Babu MM. 2004. GenCompass: A universal system for analysing gene expression for any genome. *Trends Biotechnol* **22:** (11) 552.

Ma HW, Zhao XM, Yuan YJ, Zeng AP. 2004. Decomposition of metabolic network into functional modules based on the global connectivity structure of reaction graph. *Bioinformatics* **20:** (12) 1870.

Mansourian R, Mutch DM, Aubert J, Fogel P, Le Goff JM, Moulin J, Petrov A, Rytz A, Voegel JJ, Roberts MA. 2004. The Global Error Assessment (GEA) model for the selection of differentially expressed genes in microarray data. *Bioinformatics* **20:** (16) 2726.

Marsden B, Abagyan R. 2004. SAD - A normalized structural alignment database: Improving sequence structure alignments. *Bioinformatics* **20:** (15) 2333.

Marshall OJ. 2004. PerlPrimer: Cross-platform, graphical primer design for standard, bisulphite and real-time PCR. *Bioinformatics* **20:** (15) 2471.

McAuliffe JD, Pachter L, Jordan MI. 2004. Multiple-sequence functional annotation and the generalized hidden Markov phylogeny. *Bioinformatics* **20:** (12) 1850.

Miguel RN. 2004. Sequence patterns derived from the automated prediction of functional residues in structurally-aligned homologous protein families. *Bioinformatics* **20:** (15) 2380.

Mungall CJ. 2004. Obol: integrating language and meaning in bio-ontologies. *Comp Funct Genom* **5:** (6-7) 509.

O'Donoghue SI, Meyer JEW, Schafferhans A, Fries K. 2004. The SRS 3D module: Integrating structures, sequences and features. *Bioinformatics* **20:** (15) 2476.

Parkinson H, Aitken S, Baldock RA, Bard JBL, Burger A, Hayamizu TF, Rector A, Ringwald M, Rogers J, Rosse C, Stoeckert CJ, Davidson D. 2004. The SOFG Anatomy Entry List (SAEL): An annotation tool for functional genomics data. *Comp Funct Genom* **5:** (6-7) 521.

Pasquier C, Girardot F, De Fombelle KJ, Christen R. 2004. THEA: Ontology-driven analysis of microarray data. *Bioinformatics* **20:** (16) 2636.

Perez-Enciso M, Misztal I. 2004. Qxpak: A versatile mixed model application for genetical genomics and QTL analyses. *Bioinformatics* **20:** (16) 2792.

Pieler R, Sanchez-Cabo F, Hackl H, Thallinger GG, Trajanoski Z. 2004. ArrayNorm: Comprehensive normalization and analysis of microarray data. *Bioinformatics* **20:** (12) 1971.

Porollo AA, Adamczak R, Meller J. 2004. POLYVIEW: A flexible visualization tool for structural and functional annotations of proteins. *Bioinformatics* **20:** (15) 2460.

Qu Y, Xu SZ. 2004. Supervised cluster analysis for microarray data based on multivariate Gaussian mixture. *Bioinformatics* **20:** (12) 1905.

Querec TD, Stoyanova R, Ross E, Patriotis C. 2004. A novel approach for increasing sensitivity and correcting saturation artifacts of radioactively labeled cDNA arrays. *Bioinformatics* **20:** (12) 1955.

Raphael B, Zhi DG, Tang HX, Pevzner P. 2004. A novel method for multiple alignment of sequences with repeated and shuffled elements. *Genome Res* **14:** (11) 2336.

Robes V, Larranaga P, Pena JM, Menasalvas E, Perez MS, Herves V, Wasilewska A. 2004. Bayesian network multi-classifiers for protein secondary structure prediction. *Artif Intell Med* **31:** (2) 117.

Saunders NFW, Curmi PMG, Cavicchioli R. 2004. An online database for the detection of novel archaeal sequences in human ESTs. *Bioinformatics* **20:** (15) 2361.

Schlueter JA, Dixon P, Granger C, Grant D, Clark L, Doyle JJ, Shoemaker RC. 2004. Mining EST databases to resolve evolutionary events in major crop species. *Genome* **47:** (5) 868.

Seo J, Bakay M, Chen YW, Hilmer S, Shneiderman B, Hoffman EP. 2004. Interactively optimizing signal-to-noise ratios in expression profiling: Project-specific algorithm selection and detection *p*-value weighting in Affymetrix microarrays. *Bioinformatics* **20:** (16) 2534.

Smid M, Dorssers LCJ. 2004. GO-Mapper: functional analysis of gene expression data using the expression level as a score to evaluate Gene Ontology terms. *Bioinformatics* **20:** (16) 2618.

Smith K, Hallett M. 2004. Towards quality control for DNA microarrays. *J Comput Biol* **11:** (5) 945.

Sontag E, Kiyatkin A, Kholodenko BN. 2004. Inferring dynamic architecture of cellular networks using time series of gene expression, protein and metabolite data. *Bioinformatics* **20:** (12) 1877.

Steinhauser D, Junker BH, Luedemann A, Selbig J, Kopka J. 2004. Hypothesis-driven approach to predict transcriptional units from gene expression data. *Bioinformatics* **20:** (12) 1928.

Suyama M, Torrents D, Bork P. 2004. BLAST2GENE: A comprehensive conversion of BLAST output into independent genes and gene fragments. *Bioinformatics* **20:** (12) 1968.

Tenney AE, Brown RH, Vaske C, Lodge JK, Doering TL, Brent MR. 2004. Gene prediction and verification in a compact genome with numerous small introns. *Genome Res* **14:** (11) 2330.

Tu K, Yu H, Guo Z, Li X. 2004. Learnability based further prediction of gene functions in Gene Ontology. *Genomics* **84:** (6) 971.

Walker PR, Smith B, Liu QY, Famili AF, Valdes JJ, Liu DY, Lach B. 2004. Data mining of gene expression changes in Alzheimer brain. *Artif Intell Med* **31:** (2) 137.

Wang J, Li KB, Sung WK. 2004. G-PRIMER: Greedy algorithm for selecting minimal primer set. *Bioinformatics* **20:** (15) 2473.

Wang J, Ma JZ, Li MD. 2004. Normalization of cDNA microarray data using wavelet regressions. *Comb Chem High Throughput Scr* **7:** (8) 783.

Weber G, Vinterbo S, Ohno-Machado L. 2004. Multivariate selection of genetic markers in diagnostic classification. *Artif Intell Med* **31:** (2) 155.

Williams AG, Williams RW. 2004. GenomeMixer: A complex genetic cross simulator. *Bioinformatics* **20:** (15) 2491.

Wong PWH, Lam TW, Lu N, Ting HF, Yiu SM. 2004. An efficient algorithm for optimizing whole genome alignment with noise. *Bioinformatics* **20:** (16) 2676.

Yamamoto S, Asanuma T, Takagi T, Fukuda KI. 2004. The Molecule Role Ontology: An ontology for annotation of signal transduction pathway molecules in the scientific literature. *Comp Funct Genom* **5:** (6-7) 528.

Yang ZR. 2004. Mining gene expression data based on template theory. *Bioinformatics* **20:** (16) 2759.

Zhang SD, Gant TW. 2004. A statistical framework for the design of microarray experiments and effective detection of differential gene expression. *Bioinformatics* **20:** (16) 2821.

Zhang Y, Eberhard DA, Frantz GD, Dowd P, Wu TD, Zhou Y, Watanabe C, Luoh SM, Polakis P, Hillan KJ *et al*. 2004. GEPIS-quantitative gene expression profiling in normal and cancer tissues. *Bioinformatics* **20:** (15) 2390.

Copyright © 2005 John Wiley & Sons, Ltd. 
